# Sleep bruxism: the complexity of a definitive diagnosis – case report

**DOI:** 10.1080/07853890.2021.1897389

**Published:** 2021-09-28

**Authors:** Maria Braz de Oliveira, André Almeida, Sérgio Félix, João Rua, Pedro Cebola, Catarina Godinho

**Affiliations:** a Instituto Universitário Egas Moniz (IUEM), Egas Moniz Cooperativa de Ensino Superior, Caparica, Portugal; b Centro de Investigação Interdisciplinar Egas Moniz (CiiEM), Egas Moniz Cooperativa de Ensino Superior, Caparica, Portugal

## Abstract

**Introduction:**

Bruxism is defined as a repetitive jaw-muscle activity characterised by clenching or grinding of the teeth and/or by bracing or thrusting of the mandible [1]. Its effects can be deleterious to the oral tissues and restorations, which highlights the importance of insight towards the fundamental aspects of occlusion in each patient. Dentists should therefore study and examine the individual occlusal schemes in order to plan and treat these patients [2].

**Materials and methods:**

Patient, male, 22 years old, with tooth wear compatible with bruxism. The diagnosis was made based on a multiple level of sensibility determined by the 2018 Bruxism Consensus of possible, probable or definitive diagnosis of bruxism. We applied a specific sleep bruxism questionnaire [3] plus a clinical examination and questionnaire about clinical signs and symptoms based on the Diagnostic Criteria for Temporomandibular Disorders [4]. After we applied an intra oral red coloured device for evaluation of bruxism during sleep for two, Bruxchecker®, and at the same time the patient slept with an electromyography device in the temporal muscle called Grindcare® with recording of audio and video during sleep. All the assumptions of the Helsinki Declaration have been fulfilled and an informed consent for clinical case of Clinica Dentária Egas Moniz approved by the ethic commission of Instituto Universitário Egas Moniz.

**Results:**

We have a positive diagnose for definitive bruxism confirmed with 15.6 grindings/clenching bursts per hour on the first night and 4.7 grindings/clenching bursts per hour on the second night, with audio and video we could have the perception of sounds compatible with problems of the respiratory system but absence of sounds and images compatible with tooth grinding. Clinically signs of tooth attrition were observed as well as tongue and cheek indentations, our patient also answered positively to the specific sleep bruxism questionnaire. The Bruxchecker® was helpful to see the dental wear movements.

**Discussion and conclusions:**

Polysomnography is the gold standard for the diagnosis of sleep bruxism. However, electromyography supplemented with audio and video recordings is increasingly advocated as an equally valid method. The existence of a device like Grindcare^®^ which measures the number of muscles bursts per hour associated with clinical examination allows to give a definitive bruxism diagnosis if used for a determined number of nights. Bruxchecker^®^ and Grindcare® results were somewhat confusing on both nights but this is due to extrinsic factors. The result was a definitive sleep bruxism diagnose according to the last bruxism consensus of 2018.
